# Modification of Perilla (*Perilla frutescens* (L.) Britt.) Seed Protein via Physical Treatments for Enhanced Gelation Properties

**DOI:** 10.3390/gels12090800

**Published:** 2026-09-02

**Authors:** Leyan Liu, Meixin Wang, Gaoyu Lu, Yuan Zhang, Feng Xue

**Affiliations:** 1 School of Pharmacy, Nanjing University of Chinese Medicine, Nanjing 210023, China; 18306218675@163.com (L.L.); 13089978225@163.com (M.W.); 13506272212@163.com (G.L.); 19707812822@163.com (Y.Z.); 2 Jiangsu Key Laboratory of Medicinal Substance and Utilization of Fresh Chinese Medicine, Nanjing University of Chinese Medicine, Nanjing 210023, China

**Keywords:** perilla seed protein, modification, structure, physicochemical properties, probiotic fermentation, gels, plant-based yogurt-like products

## Abstract

Perilla (*Perilla frutescens* (L.) Britt.) seed protein, an underutilized resource derived from medicinal and edible plants, was subjected to ultrasound (400 W, 20 min), pH-shifting (pH 12.0, 1 h), and high-pressure homogenization (90 MPa, 3 cycles), applied individually or in combination, and subsequently fermented with probiotics to induce gel formation. The triple combination (pH-shifting + high-pressure homogenization + ultrasound) produced the highest solubility increase (129%), smallest particle size, greatest random coil content, and maximal surface hydrophobicity and free sulfhydryl level. The triple-modified protein gels exhibited a highly homogeneous microstructure. Meanwhile, these gels achieved significant improvements (*p* < 0.05) in multiple functional and rheological properties, including gel strength, water-holding capacity, viscosity, and storage modulus, as well as the viable count. These results demonstrate that combined application of three physical modifications effectively restructures perilla seed protein and markedly improves its gelation behavior under fermentation, supporting its potential as a novel ingredient for fermented plant-protein gels or yogurt-like products.

## 1. Introduction

Driven by the urgent need to mitigate the environmental footprint of animal agriculture and to ensure food security for a growing global population, the food industry is actively seeking sustainable protein alternatives [1]. Plant-derived proteins, owing to their lower resource consumption and compatibility with diverse dietary practices, have emerged as promising candidates to replace animal-based ingredients in various food applications. Proteins from soy [2], pea [3], chickpea [4], lentil [5], and other cereal sources [6] have demonstrated potential as replacements for animal proteins in food systems. However, one significant protein source has long been overlooked: medicinal and edible plant resources in China. Many of these resources are rich in proteins, yet they are typically exploited for the extraction of phenolic compounds, oils, and polysaccharides, with the protein fraction being discarded as a byproduct. This leads to a considerable waste of protein resources. In recent years, research on proteins derived from medicinal and edible plant resources has gradually attracted attention. For instance, hemp seed proteins have been found to possess both functionality and bioactivity, as various bioactive peptides have been isolated from them [7]. However, the widespread adoption of plant proteins is often constrained by their inferior techno-functionality compared to animal-derived counterparts. This is largely attributed to their compact, rigid molecular structures and poor solubility, which limit their performance in processes like gelation and emulsification. Consequently, various physical, chemical, or biological modification strategies have been explored to improve these functional properties.

Physical techniques are widely employed to improve the functional properties of plant proteins by altering their structural conformations. Ultrasound treatment, for instance, generates acoustic cavitation forces that disrupt non-covalent bonds and induce protein unfolding, thereby exposing internal groups and enhancing functionality [8]. Studies have shown that ultrasound can effectively improve the solubility, emulsifying, and foaming properties of various plant proteins [9]. High-pressure homogenization subjects protein dispersions to intense shear, turbulence, and cavitation, leading to disruption of aggregated structures with altered properties [10]. High-pressure homogenization has been reported to improve the solubility, foaming, and emulsification capacities of proteins [11]. The pH-shifting method, which involves exposing proteins to highly acidic or alkaline conditions followed by neutralization, promotes partial unfolding and refolding, thereby exposing buried reactive groups and increasing protein flexibility [12]. This technique has been successfully applied to enhance the functionality of various plant proteins [13]. Recent studies show that combining these methods yields pronounced effects: pH-shifting plus ultrasound improved walnut protein properties [14]; pH-shifting plus homogenization enhanced pea protein functionality [15]; and homogenization coupled with ultrasound increased hemp protein solubility [16]. Thus, combined modification strategies represent a promising approach for improving plant protein functionality.

Plant-based foods, particularly yogurt, are gaining market traction [17]. While commercial plant-based yogurts predominantly use soy, oat, pea, and rice proteins, proteins from medicine-food homology resources like plum [18] and hemp seeds [19] have recently shown promise for probiotic fermentation-induced gelation. Perilla (*Perilla frutescens* (L.) Britt.) seed represents a valuable yet underexplored resource, currently utilized primarily for oil [20] and polysaccharide [21] extraction, while their protein fraction remains largely uncharacterized.

Therefore, this study aims to modify perilla seed protein using three techniques, namely high-pressure homogenization, pH-shifting, and ultrasound treatment, applied either individually or in binary and ternary combinations. The molecular mechanisms underlying the modification will be investigated. Subsequently, the modified perilla seed protein will be subjected to probiotic fermentation, and the properties of the resulting gels will be characterized. The goal is to elucidate how the modification techniques influence gelation behavior, thereby providing a scientific basis for the application of perilla seed protein in fermented plant-protein gels intended for yogurt-like applications.

## 2. Results and Discussion

### 2.1. Solubility of Perilla Seed Protein Isolates (PSPI)

**Figure 1:**
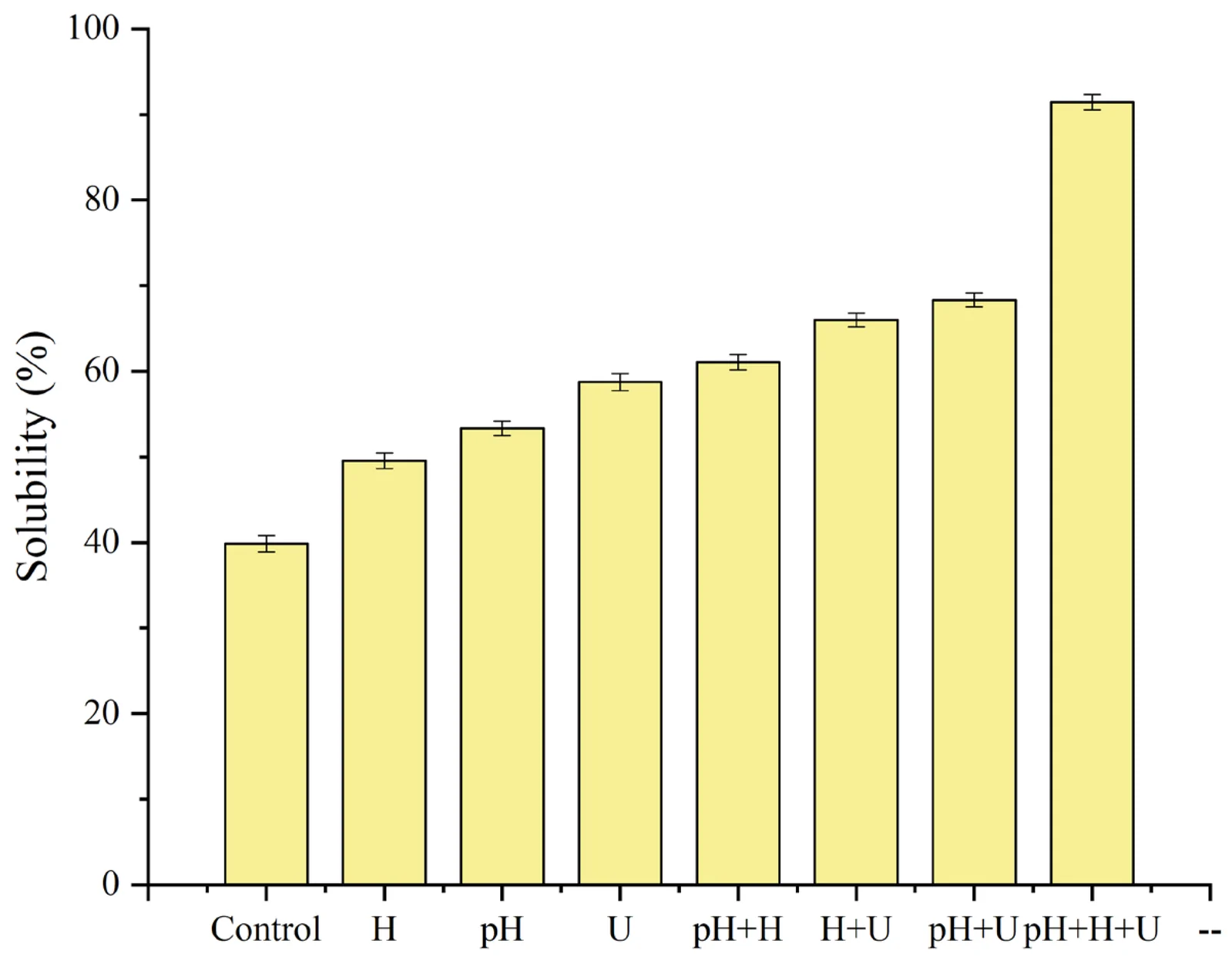
Influence of high-pressure homogenization, pH-shifting, and ultrasound on the solubility of perilla seed protein. The abbreviation codes for each treatment are defined as follows: U (ultrasound only), pH (pH-shifting only), H (high-pressure homogenization only), pH+U (pH-shifting plus ultrasound), H+U (high-pressure homogenization plus ultrasound), pH+H (pH-shifting plus high-pressure homogenization), and pH+H+U (pH-shifting, high-pressure homogenization, and ultrasound combined). Data represent the mean ± SD of three technical replicates performed on the same independently prepared PSPI batch and are presented to illustrate measurement precision. No statistical inference was drawn from these data.

Figure 1 shows that each of the three modification treatments enhanced PSPI solubility. This improvement is primarily ascribed to the conformational rearrangements induced by these physical interventions. Processes like ultrasound (U) cavitation, high-pressure (H) shearing, and pH-shifting (pH) all effectively disrupt the native, compact protein aggregates. This disruption leads to a reduction in particle size [12] and the unfolding of the protein structure [22], as evidenced by the increased random coil content and altered tertiary conformations (discussed in Section 2.5). Consequently, previously buried hydrophobic patches and reactive side chains become exposed on the protein surface [23]. This enhances protein-water interactions, weakens protein–protein interactions, and ultimately results in a marked increase in solubility and dispersibility. Notably, among the three individual treatments, ultrasound produced the most pronounced enhancement in PSPI solubility. This trend also influenced the combined treatments: any binary combination that included ultrasound outperformed those without it. Among all the modifications, the triple combination (pH+H+U) exhibited the greatest effect among all tested sequences, resulting in a 129% increase in solubility. Based on the solubility screening results, we adopted a tiered selection strategy for subsequent structural and functional characterization. Specifically, among the three individual treatments, ultrasound (U) exhibited the most pronounced enhancement in PSPI solubility and was therefore chosen as the representative single modification. Among the binary combinations, the pH-shifting plus ultrasound (pH+U) group showed the greatest improvement and was selected to represent the two-step modifications. Finally, the triple combination (pH+H+U) was included as it consistently outperformed all other groups across the solubility measurements.

### 2.2. Microstructure of PSPI by Transmission Electron Microscopy (TEM)

**Figure 2:**
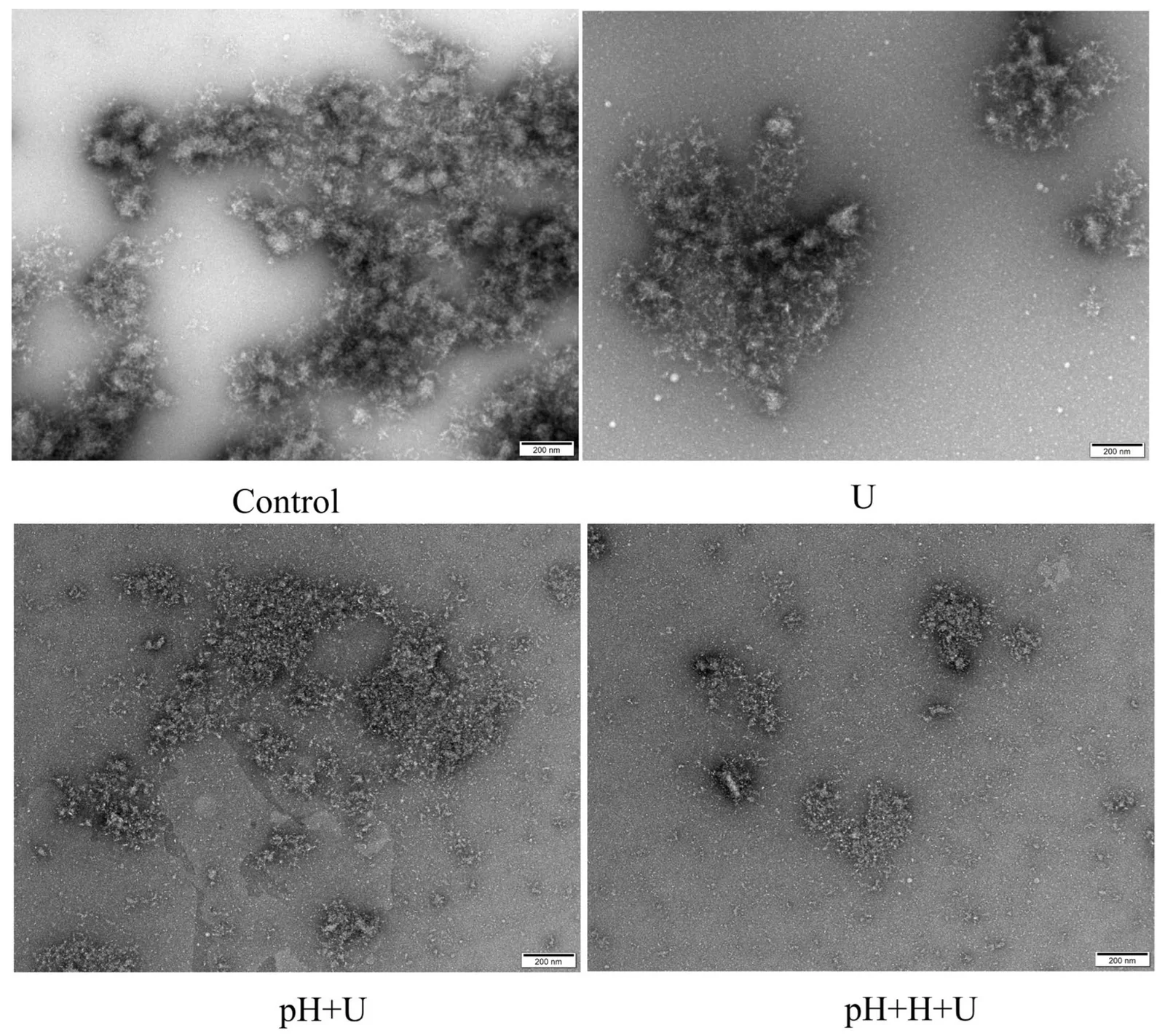
TEM images of perilla seed protein under various modification conditions. Treatment labels are the same as in Figure 1.

As depicted in TEM images (Figure 2), native PSPI appeared as spherical particles with diameters ranging from 100 to 200 nm. This observation is consistent with earlier reports on native proteins [24]. Moreover, these spherical particles tended to aggregate into large clusters, which explains the poor solubility of native PSPI. After ultrasound treatment, the large aggregates were visibly broken down into smaller ones, accompanied by the appearance of some spherical particles of even smaller size. This effect became more pronounced with the combination of pH-shifting and ultrasound: the original 100–200 nm spherical particles nearly disappeared and were replaced by much finer particles; nevertheless, these smaller particles still existed in aggregated forms. When the triple modification (pH+H+U) was applied, the degree of aggregation among the small particles was markedly reduced. This observation provides a clear explanation for why this combined treatment yielded the highest solubility.

### 2.3. Particle Size Distribution and Zeta Potential of PSPI

**Figure 3:**
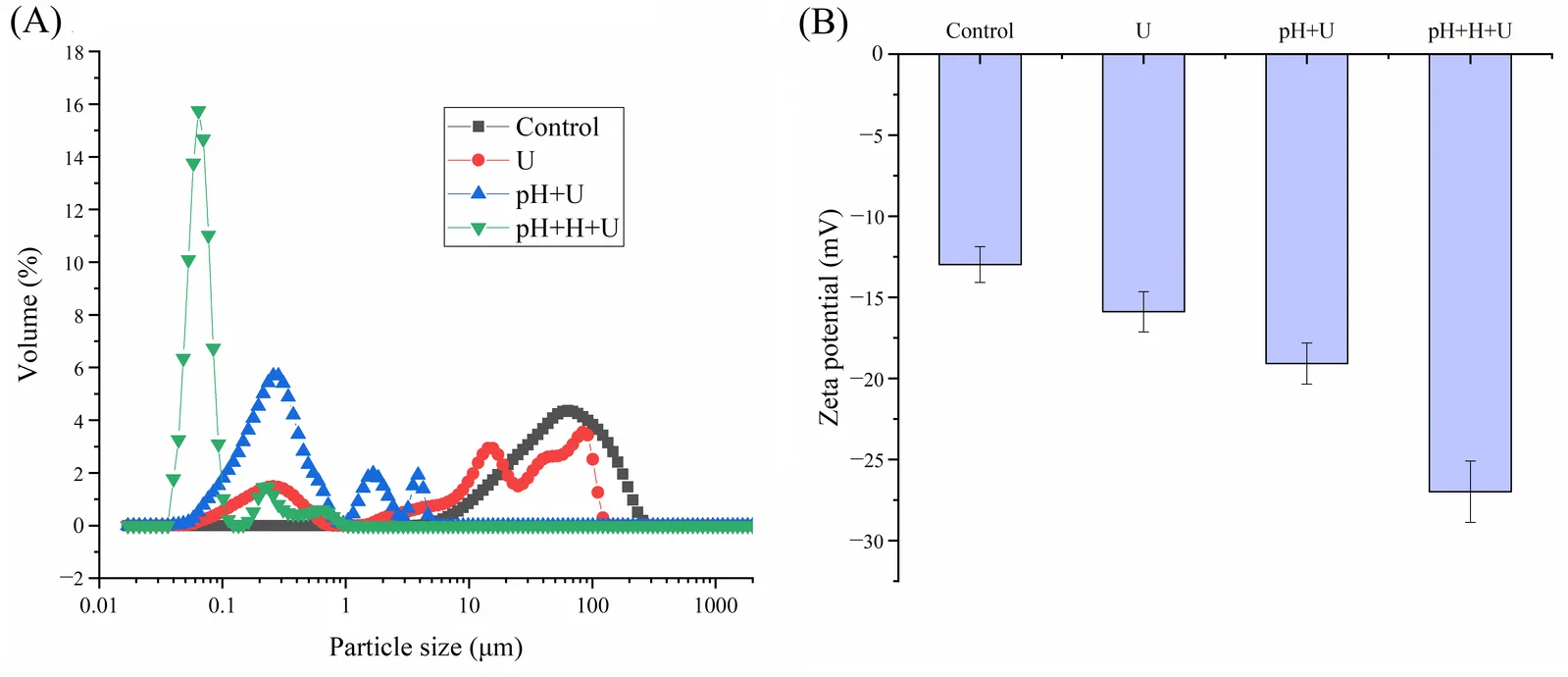
Particle size distribution (**A**) and zeta potential (**B**) of perilla seed protein following high-pressure homogenization, pH-shifting, and ultrasound treatments. Treatment labels are the same as in Figure 1. Data represent the mean ± SD of three technical replicates performed on the same independently prepared PSPI batch and are presented to illustrate measurement precision. No statistical inference was drawn from these data.

Figure 3A reveals that the unmodified PSPI displayed a dominant particle size population centered at roughly 100 μm, again confirming that it existed primarily as large aggregates, consistent with the TEM observations. After ultrasound treatment, the proportion of particles at the original 100 μm peak began to decrease, while new peaks emerged at approximately 10 μm and even smaller sizes, indicating that ultrasound reduced the degree of aggregation of native PSPI. Consistent with earlier observations on almond protein isolate, ultrasound treatment disrupts protein aggregates, thereby decreasing particle size and improving solubility [25]. Upon the combined application of pH-shifting and ultrasound, the large distribution peak disappeared and was replaced by a dominant peak at around 0.2 μm, which agreed well with the TEM findings. This suggests that the combined treatment not only disrupted large aggregates but also reduced the size of the individual protein particles. Studies on walnut protein have similarly found that pH-shifting combined with ultrasound outperformed each individual treatment in reducing particle size [14]. The greater reduction in particle size observed for the pH+U sequence compared with U alone may be partly explained by the fact that alkaline pretreatment renders the protein more susceptible to subsequent ultrasonic cavitation forces. However, because detailed characterization was limited to the selected sequences, this mechanistic interpretation remains speculative. As a result, a greater number of non-covalent bonds are disrupted, and the large protein clusters are dissociated into smaller fragments. Furthermore, when triple modification (pH+H+U) was applied, PSPI exhibited the smallest particle size, again consistent with TEM observations. This indicates that following pH-shifting and high-pressure homogenization, the protein becomes even more sensitive to the cavitation effects of ultrasound, leading to an additional decrease in particle size.

As shown in Figure 3B, ultrasonic treatment increased the net surface charge of PSPI. This effect may be attributed to the ultrasound-induced unfolding of the protein structure, which simultaneously exposes previously buried charged amino acid residues on the protein surface, thereby increasing the absolute zeta potential [26]. The increased negative surface charge consequently boosted electrostatic repulsion among neighboring protein molecules [27], which could explain the reduced protein aggregation observed in the TEM images. When PSPI was subjected to combined pH-shifting and ultrasound treatment, the net surface charge increased further. The further unfolding of PSPI structure is likely attributable to the combined influence of both treatments, which simultaneously modify protein conformation and surface-exposed groups [28]. Moreover, the highest net surface charge was achieved when all three methods were applied, indicating that high-pressure homogenization further enhanced the effects of the other two treatments. Previous studies have demonstrated that high-pressure homogenization facilitates additional rearrangement of the amino acid surface landscape after protein unfolding [29], thus leading to an elevation in the absolute zeta potential.

### 2.4. Subunits of PSPI

**Figure 4:**
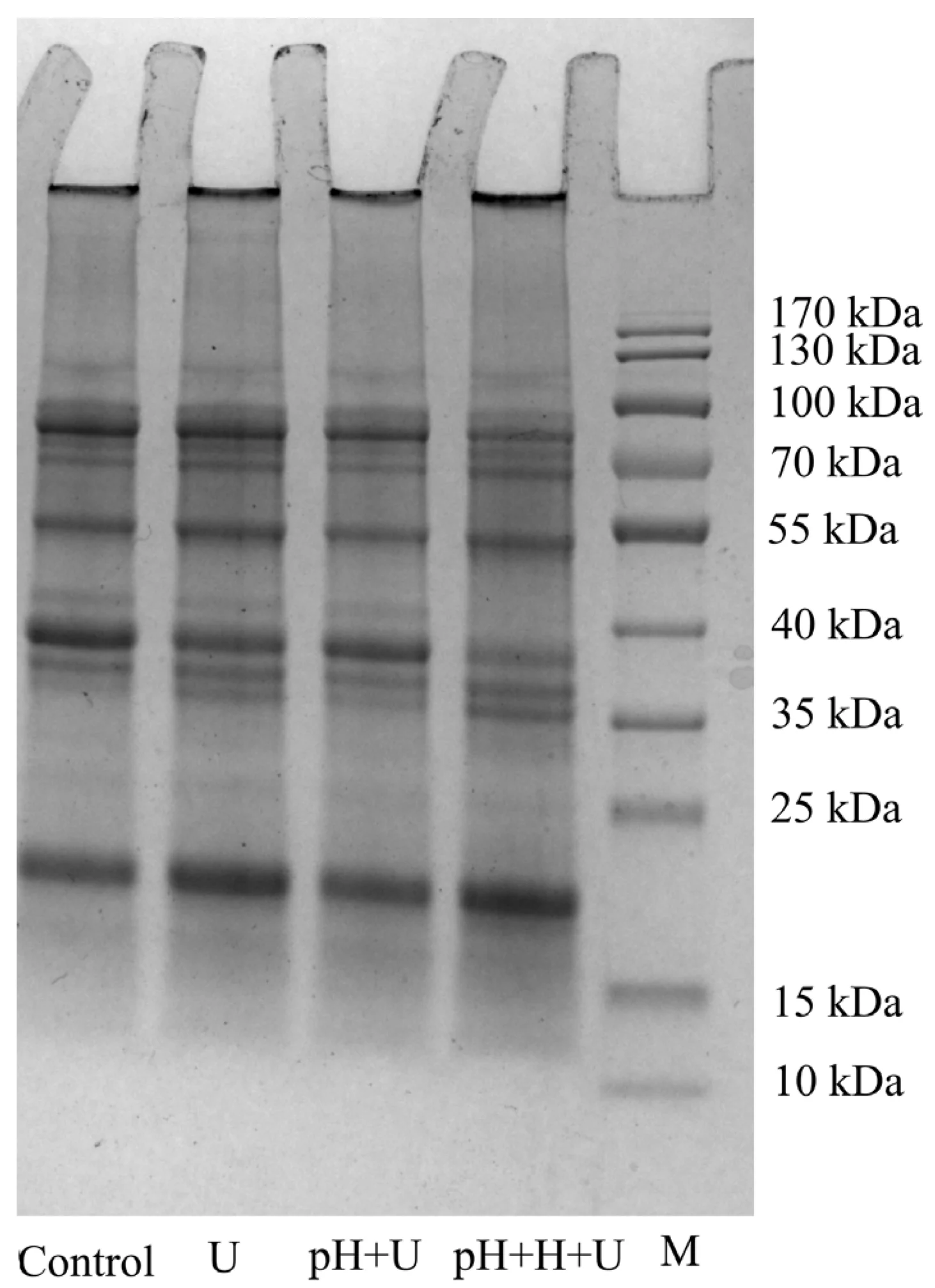
Electrophoretic profiles of perilla seed protein subjected to high-pressure homogenization, pH-shifting, and ultrasound treatments. Treatment labels are the same as in Figure 1.

The SDS-PAGE profiles of the U, pH+U, and pH+H+U samples presented in Figure 4 showed no discernible differences from those of the unmodified PSPI. This indicates that ultrasound, whether applied alone or in combination with pH-shifting treatment, did not affect the major protein subunits nor cause their degradation into smaller fragments under reducing conditions. Similarly, the triple combination (pH+H+U) also left the protein subunits unchanged. These findings corroborate earlier work showing that ultrasound treatment, pH-shifting, high-pressure homogenization, or any binary combination of these three modification methods had no discernible effect on protein subunit composition [8,15,30].

### 2.5. Secondary and Tertiary Structure Analysis of PSPI

**Figure 5:**
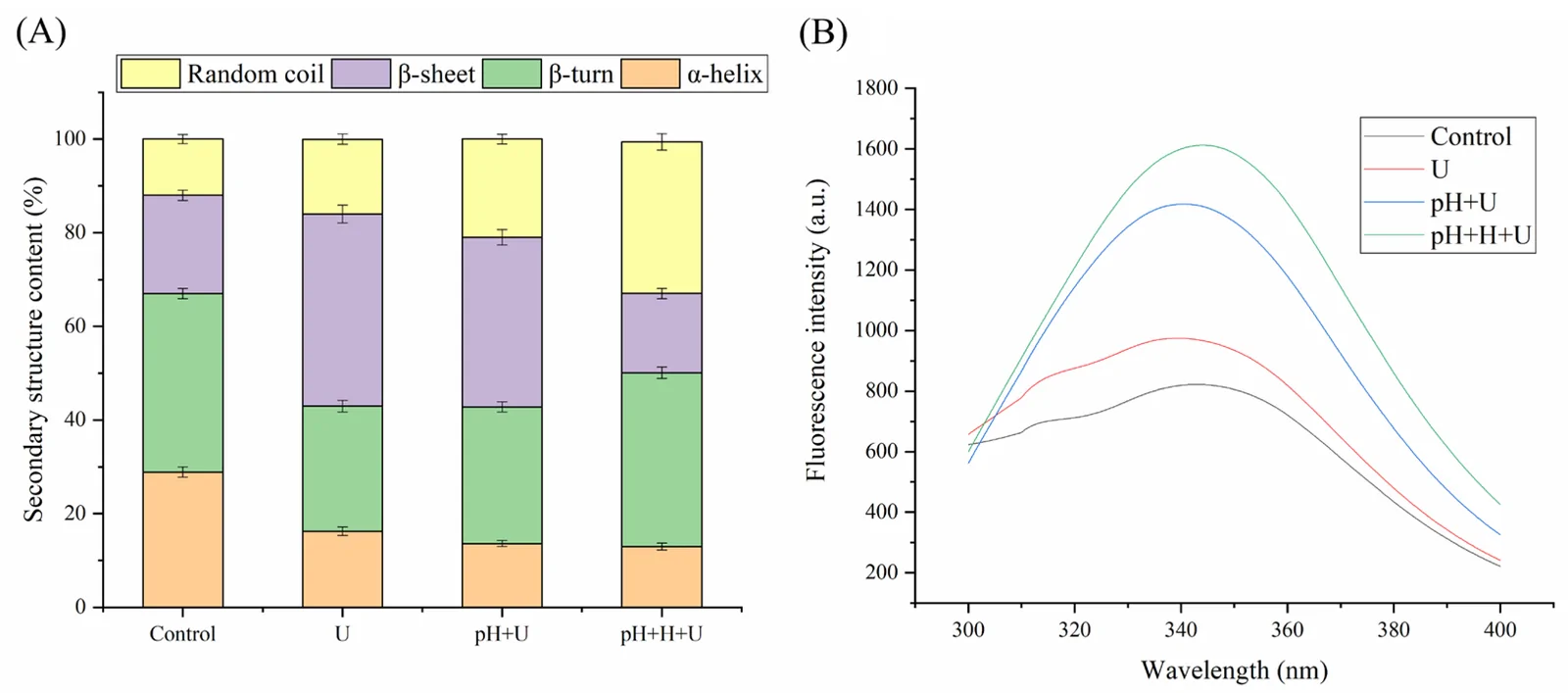
Conformational changes in perilla seed protein at the secondary (**A**) and tertiary (**B**) structural levels induced by high-pressure homogenization, pH-shifting, and ultrasound. Treatment labels are the same as in Figure 1. Data represent the mean ± SD of three technical replicates performed on the same independently prepared PSPI batch and are presented to illustrate measurement precision. No statistical inference was drawn from these data.

The random-coil fraction in the secondary structure of PSPI was elevated following ultrasonic treatment (Figure 5A). This conformational shift is largely ascribable to the cavitation forces generated during sonication, which weaken the hydrogen-bonding network and drive the protein toward more flexible, less ordered states. Consequently, the structural rigidity of the protein is diminished [31]. Such conformational unfolding generally exposes hydrophilic residues and polar groups to the solvent, enhancing protein-water interactions and thus contributing to improved solubility [32]. This structural interpretation is in good agreement with the solubility results presented in Figure 1, where a marked increase in solubility was observed for the same modified samples. When PSPI was subjected to combined pH-shifting and ultrasound treatment, the random coil content increased further. This combined effect is likely attributable to the enhanced disruption of hydrogen bonds by the dual treatment, which drives the protein structure from an ordered, compact state toward a more disordered conformation. Moreover, the increased formation of random coils reflects a tendency toward greater disorder and conformational entropy. The beneficial effect of such combined treatment on solubility has been linked to several molecular events, including the reduction of hydrogen bonding and electrostatic interactions on the protein surface, the disruption of its rigid conformation, and the enhancement of molecular flexibility [28]. Furthermore, the highest random coil content was observed when all three methods were applied together. This suggests that the addition of high-pressure homogenization further promotes protein unfolding. The application of mechanical shear during homogenization has been reported to disrupt intramolecular hydrogen bonds, thereby inducing structural rearrangement of ordered protein domains [33]. This is followed by the breakdown of non-covalent interactions under high-pressure, which initiates unfolding and subsequent disulfide bond cleavage. The conformational change thereby exposes hydrophobic residues, facilitating protein-water interactions and ultimately resulting in enhanced solubility [34]. This may also explain why the combination of all three treatments yielded the highest protein solubility.

The intrinsic fluorescence intensity at the maximum emission wavelength was notably enhanced following ultrasonic treatment (Figure 5B). This increase is largely explained by the cavitation and shear stresses exerted during sonication, which perturb the native protein conformation. The resulting structural perturbations can manifest as either aggregation or disaggregation of the protein molecules [35]. As a result of these structural alterations, tryptophan residues and hydrophobic groups become more exposed, giving rise to higher fluorescence intensity corresponding to the maximum emission wavelength [36]. A further increase in the maximum emission fluorescence intensity was observed upon the combined application of pH-shifting and ultrasound (pH+U) compared with ultrasound alone, indicating that the sequential treatment induced additional tertiary conformational changes that shifted buried aromatic side chains into a more polar environment. This observation is consistent with earlier reports [14,28], in which the increased fluorescence was interpreted as a consequence of tertiary conformational changes that shift buried aromatic side chains into a more polar environment. When all three treatments were applied simultaneously, the fluorescence intensity at the emission maximum reached its highest level. This result suggests that high-pressure homogenization also contributes to promoting protein unfolding [37].

### 2.6. Surface Hydrophobicity (H_0_), Thermal Denaturation and Free Sulfhydryl (SH) Content of PSPI

The surface hydrophobicity of PSPI was increased upon ultrasonic treatment. This enhancement is ascribed to the partial breakdown of native protein aggregates under cavitation forces, which exposes previously inaccessible hydrophobic moieties (such as methyl groups and aromatic residues) to the protein surface [38]. The combination of pH-shifting and ultrasound further enhanced the surface hydrophobicity of PSPI. This is mainly because the pH-shifting treatment disrupts the protein structure under alkaline conditions, while ultrasonic cavitation further dissociates the protein, collectively leading to greater exposure of hydrophobic groups on the protein surface [36]. The triple combination of all three methods yielded the greatest surface hydrophobicity. This result indicates that adding high-pressure homogenization to the previous two treatments promotes further exposure of hydrophobic groups within the protein. The increase in surface hydrophobicity induced by high-pressure homogenization has been linked to the penetration of water molecules into the protein core and the concomitant weakening of intermolecular hydrophobic contacts [29].

**Figure 6:**
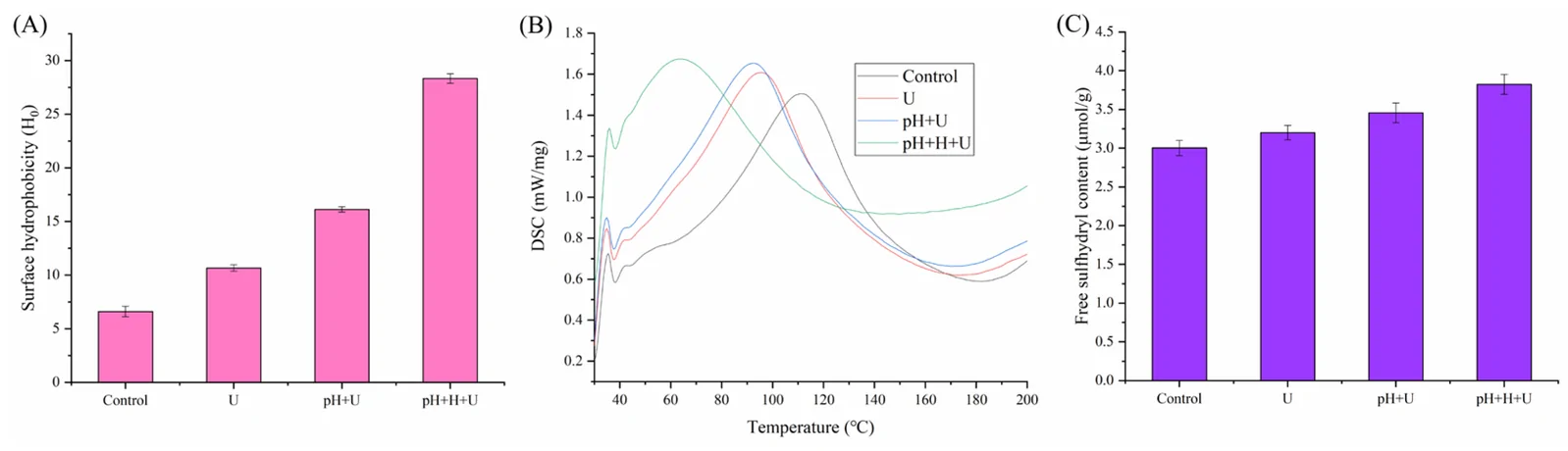
Influence of high-pressure homogenization, pH-shifting, and ultrasound on the surface hydrophobicity (**A**), thermal denaturation (**B**), and free sulfhydryl content (**C**) of perilla seed protein. Treatment labels are the same as in Figure 1. Data represent the mean ± SD of three technical replicates performed on the same independently prepared PSPI batch and are presented to illustrate measurement precision. No statistical inference was drawn from these data.

Figure 6B shows that ultrasonic treatment reduced the thermal denaturation temperature of PSPI. This observation aligns with previous reports, in which the cavitation effect of ultrasound disrupted protein interactions, thereby lowering the thermal denaturation temperature of protein [39]. When PSPI was treated with a combination of pH-shifting and ultrasound, the thermal denaturation temperature decreased further. Moreover, the lowest thermal denaturation temperature was achieved when all three methods were applied together. These results again confirm a combined effect among the three treatments, which further disrupts intermolecular forces or promotes protein unfolding, ultimately rendering the protein more thermally sensitive.

Figure 6C demonstrates that ultrasound treatment raised the free sulfhydryl level in PSPI. One possible explanation is that ultrasonication made previously hidden -SH groups accessible [40]. Moreover, evidence indicates that ultrasound can break disulfide bonds, which also leads to an increase in -SH content [36]. The combined application of pH-shifting and ultrasound resulted in an even higher free sulfhydryl content, presumably because ultrasonication facilitated the exposure of -SH groups buried inside the protein, especially after exposure to an alkaline environment [14]. Notably, the triple combination (pH+H+U) yielded the highest free sulfhydryl content among the three characterized groups. The sequential addition of high-pressure homogenization to pH-shifting and ultrasound thus coincided with further increases in free SH content, although the current experimental design does not permit this to be interpreted as an independent contribution of high-pressure homogenization. Previous findings have shown that high-pressure homogenization further boosts free sulfhydryl content through various phenomena, such as unfolding-driven exposure of internal groups and disulfide bond disruption, along with possible backbone fragmentation and aggregation [37]. The newly exposed or liberated -SH groups were hypothesized to serve as reactive precursors that could participate in the formation of new disulfide bonds during subsequent gelation induced by probiotic fermentation.

### 2.7. PSPI Gels Induced by Probiotic Fermentation

All gel property measurements (whiteness, pH, gel strength, WHC, and viable count) were performed on three independently prepared and fermented gel batches (*n* = 3 independent replicates), and statistical comparisons were conducted by one-way ANOVA followed by Tukey’s HSD test.

#### 2.7.1. Physicochemical Properties of PSPI Gels

**Figure 7:**
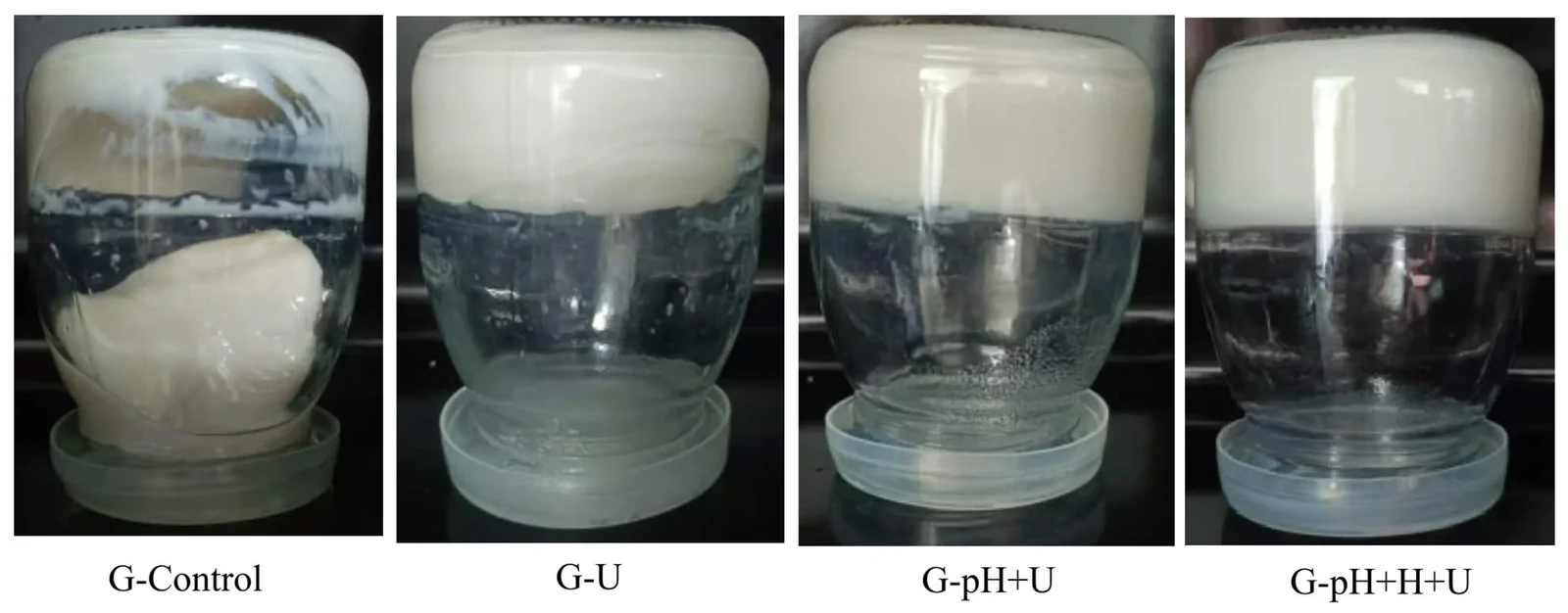
Photographs of perilla seed protein gels subjected to high-pressure homogenization, pH-shifting, and ultrasound treatments. The designations G-U, G-pH+U, and G-pH+H+U denote gels obtained from the respective modification conditions. Treatment labels are the same as in Figure 1.

As shown in Figure 7, all samples formed gels after 10 h of fermentation. This result is consistent with previous reports on plant protein gels induced by probiotic fermentation, such as those from soybean [41], hemp seed [19], and plum seed proteins [18], which were found to induce gelation within 8 to 12 h of fermentation. However, the gel prepared from native PSPI exhibited poor stability and failed to adhere to the bottle wall. In contrast, all modified PSPI samples formed stable gels. Nevertheless, the gel produced from ultrasonically modified PSPI displayed irregular morphology at the bottom and showed partial detachment from the bottle wall. This phenomenon was alleviated in the gel prepared from PSPI subjected to combined pH-shifting and ultrasound treatment. Notably, when all three modification methods were applied, the resulting PSPI gel was stable, with a smooth and uniform bottom, and showed no signs of wall separation. These findings indicate that modification promotes the formation of probiotic-induced PSPI gels, with the triple combination showing the most pronounced effect.

**Figure 8:**
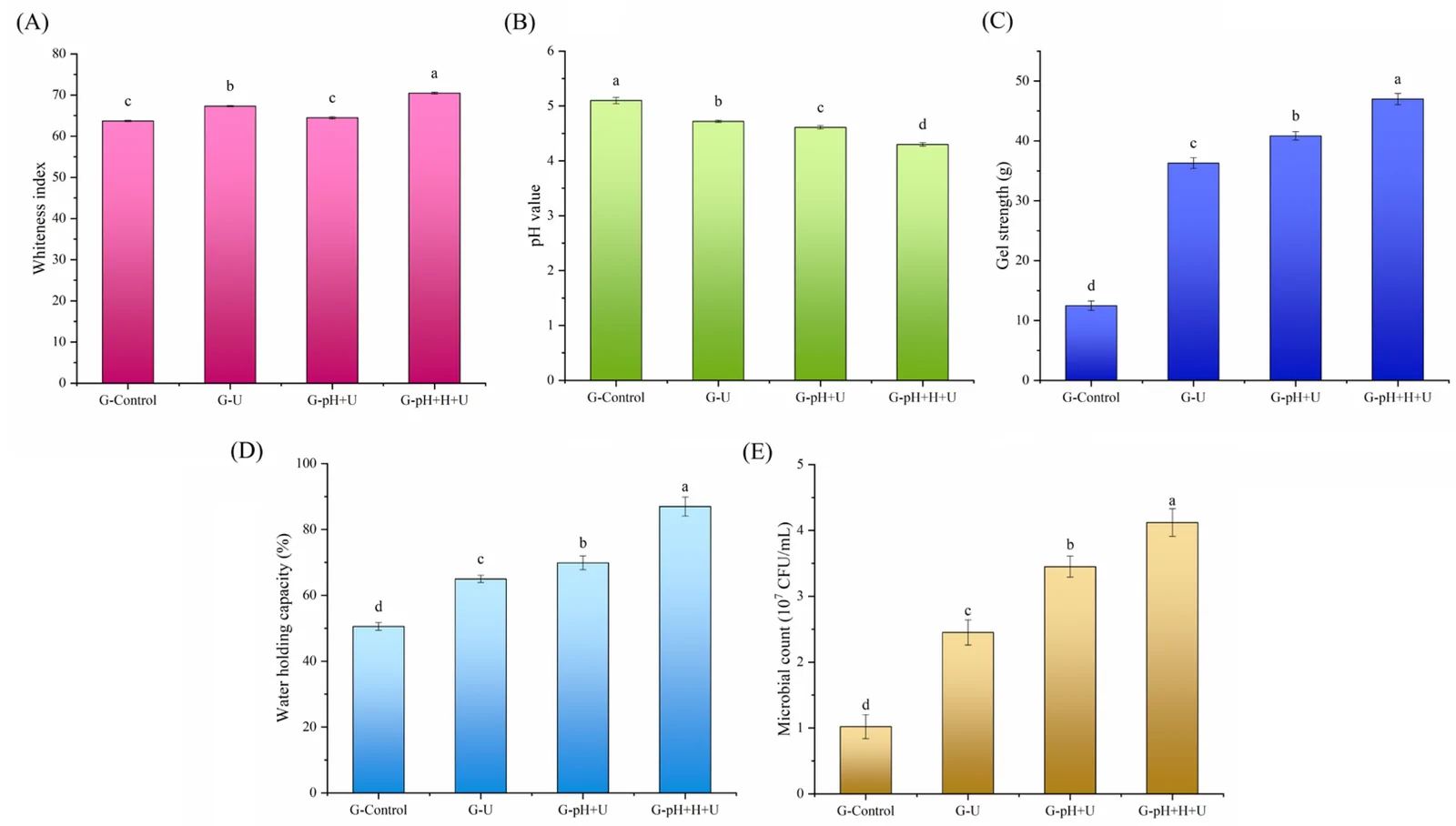
Effect of high-pressure homogenization, pH-shifting, and ultrasound treatment on whiteness index (**A**), pH value (**B**), gel strength (**C**), water holding capacity (**D**), and viable count (**E**) of perilla seed protein gels. G-U, G-pH+U, and G-pH+H+U represent protein gels prepared by different treatments, respectively. Treatment labels are the same as in Figure 1. Different lowercase letters within the same pattern indicate statistically significant differences (*p* < 0.05, *n* = 3, each replicate representing an independently prepared and fermented gel batch).

As shown in Figure 8A, ultrasonic treatment significantly (*p* < 0.05) increased the whiteness of PSPI gel. This is likely because the gel formed under ultrasound exhibited greater stability, which enhanced light reflection and consequently led to higher whiteness. When pH-shifting was combined with ultrasound, however, the whiteness of the gel decreased. A potential explanation for this decrease is the oxidation of remaining phenolic compounds in PSPI under alkaline pH [42]. Notably, when all three modification methods were applied together, the resulting PSPI gel showed the highest whiteness, which may be associated with the formation of a smooth gel surface. A smooth gel surface enhances light reflection, which is known to impart a white appearance to the gel [43].

As shown in Figure 8B, modification significantly (*p* < 0.05) reduced the final pH, indicating that gels from modified PSPI reached a lower final pH than the control. One possible explanation is that the smaller particle size and more extended conformation of the modified proteins may have provided a more accessible environment for the probiotics. However, it should be noted that the current endpoint pH and viable-count measurements alone do not directly demonstrate enhanced acid production or metabolic activity of the probiotics. Furthermore, among all samples, PSPI subjected to the triple modification method produced gels with the lowest final pH, which was closest to the isoelectric point of PSPI. Consistent with previous studies on soy [43], pea [44], and mung bean [45] proteins, it is plausible that probiotic fermentation induces PSPI gel formation through the production of organic acids, which lower the pH toward the isoelectric point of PSPI, reduce electrostatic repulsion, and thereby promote protein aggregation into a gel network. Nonetheless, while this acid-mediated mechanism is a reasonable hypothesis supported by the observed decrease in final pH and the known behavior of plant proteins in the literature, it is not directly established by the current endpoint measurements. Future studies employing real-time pH monitoring and quantification of specific organic acids would be required to confirm the acid-production kinetics and to fully validate this mechanism.

A significant improvement in gel strength was observed for modified PSPI gels (*p* < 0.05, Figure 8C), with the strongest gel achieved by the triple combination treatment. This enhancement is probably associated with the surface exposure of functional groups, including hydrophobic residues and free sulfhydryl groups, which are capable of forming hydrophobic interactions and disulfide bonds that stabilize the gel network. Moreover, the modified gels reached pH values nearer to the isoelectric point, thereby reducing electrostatic repulsion between protein molecules and promoting closer molecular associations. This further promoted the formation of intermolecular interactions, contributing to the increase in gel strength. Previous studies on plant-based yogurt have similarly highlighted the critical roles of hydrophobic interactions and disulfide bonds during gel formation [18,44].

As shown in Figure 8D, modification significantly (*p* < 0.05) improved the water-holding capacity of PSPI gels, with the highest value achieved when all three methods were combined. This enhancement can be attributed to the improved hydration capability of PSPI following modification, which is supported by the solubility measurements. Furthermore, modification appeared to promote the formation of intermolecular interactions during gelation, which is consistent with a more stable network structure and a compact microstructure, and may thereby strengthen the gel’s ability to retain water molecules. This observation is consistent with the gel strength results. A positive correlation between gel network compactness/stability and water-holding capacity has also been documented in plant-based yogurt systems [41].

As shown in Figure 8E, all gels exhibited a viable count of 1 × 10^7^ CFU/mL, which is comparable to the minimum threshold (1 × 10^7^ CFU/mL) for total viable counts in fermented dairy products as defined by the Codex Alimentarius [46], and falls within the range of 0.88–2.60 × 10^7^ CFU/mL previously reported for other fermented plant-based protein gels [41]. These findings indicate that PSPI holds promise as a candidate for formulating fermented plant-based gel products. Furthermore, modification markedly increased the viable count in PSPI-formed gels, with the highest value observed when all three modification strategies were applied together. This result is consistent with the lower final pH reached by the modified gels, which would be expected to promote protein aggregation as the pH approaches the isoelectric point, potentially contributing to the formation of a more compact protein network.

#### 2.7.2. Rheological Properties of PSPI Gels

**Figure 9:**
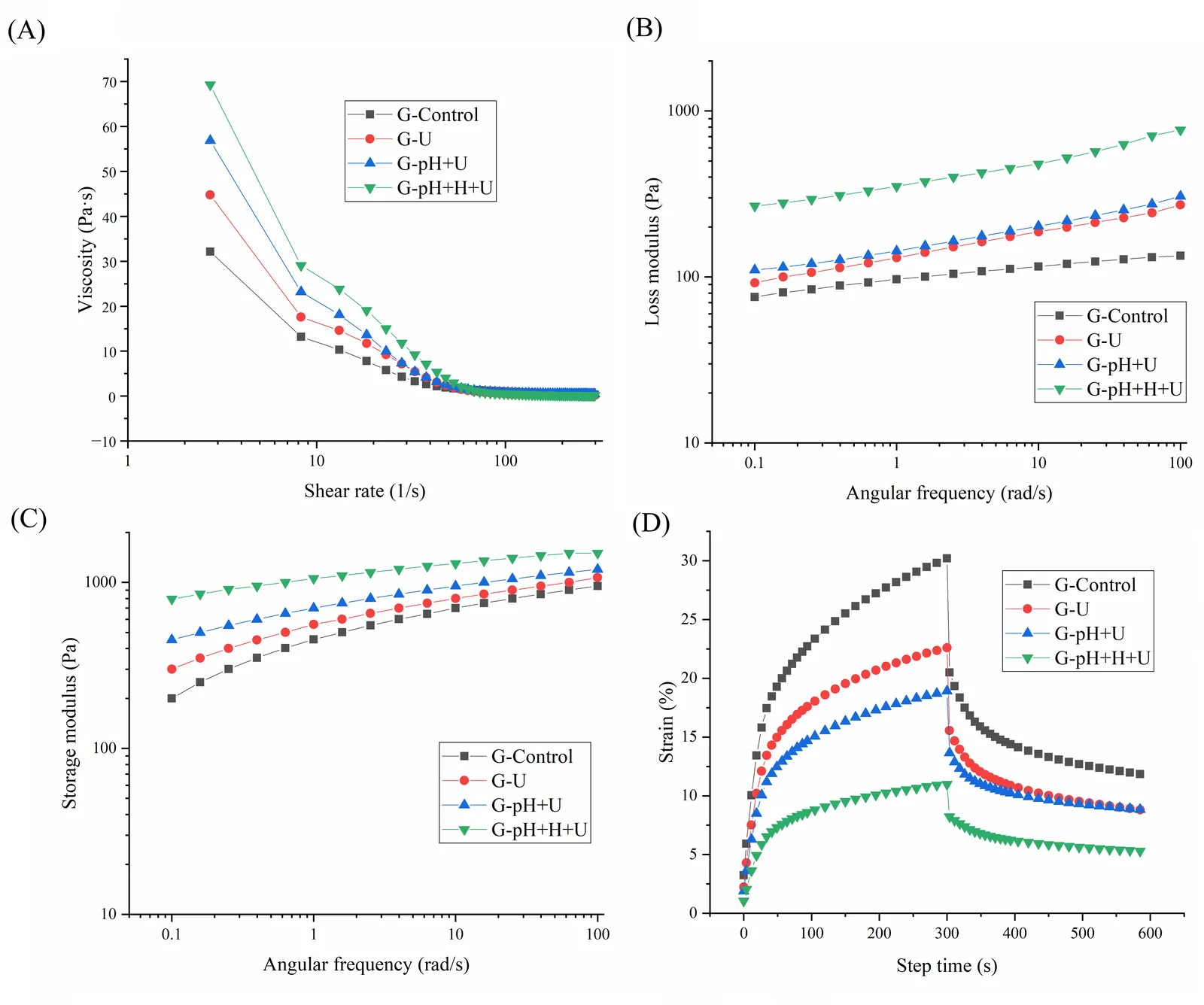
Viscosity (**A**), loss modulus (**B**), storage modulus (**C**), creep and recovery property (**D**) of perilla seed protein gels subjected to high-pressure homogenization, pH-shifting, and ultrasound treatments. G-U, G-pH+U, and G-pH+H+U represent protein gels prepared by different treatments, respectively. Treatment labels are the same as in Figure 1.

The shear-thinning behavior observed in all gel samples (Figure 9A) implies that the non-covalent bonds sustaining the gel network were compromised upon shearing [47]. It is noteworthy that gels prepared with modified PSPI displayed higher apparent viscosity than the control, with the highest value observed for gels made from PSPI subjected to all three modification methods. This result suggests that modification strengthens intermolecular interactions that maintain gel structure. These observations are in line with findings on the effects of modification on PSPI hydration and surface hydrophobicity. The enhanced water-holding capacity after modification may provide more hydrogen bonds during gel formation, whereas the increased surface hydrophobicity likely contributes additional hydrophobic interactions. Earlier studies have similarly reported that stronger intermolecular forces within a gel are associated with greater apparent viscosity [18].

As shown in Figure 9B,C, all tested samples displayed a storage modulus exceeding the loss modulus, indicating typical gel-like behavior. This observation is consistent with earlier findings reported for coconut [48] and soy milk yogurt [49]. Compared with the control group, gels prepared from modified PSPI showed higher storage moduli, with the highest value achieved by PSPI subjected to all three modification methods. These results suggest that modified PSPI forms gels with enhanced structural stability and integrity.

A deformation exceeding 30% was observed for the control gel (Figure 9D), reflecting substantial structural failure under the applied load. Such pronounced deformation is primarily ascribed to the inherently weak network architecture of the unmodified gel [50]. In contrast, gels prepared with modified PSPI showed reduced deformation, with the smallest value observed for PSPI subjected to all three modification methods. These results further demonstrate that modification helps PSPI reinforce the internal gel network.

#### 2.7.3. Microstructure of PSPI Gels

**Figure 10:**
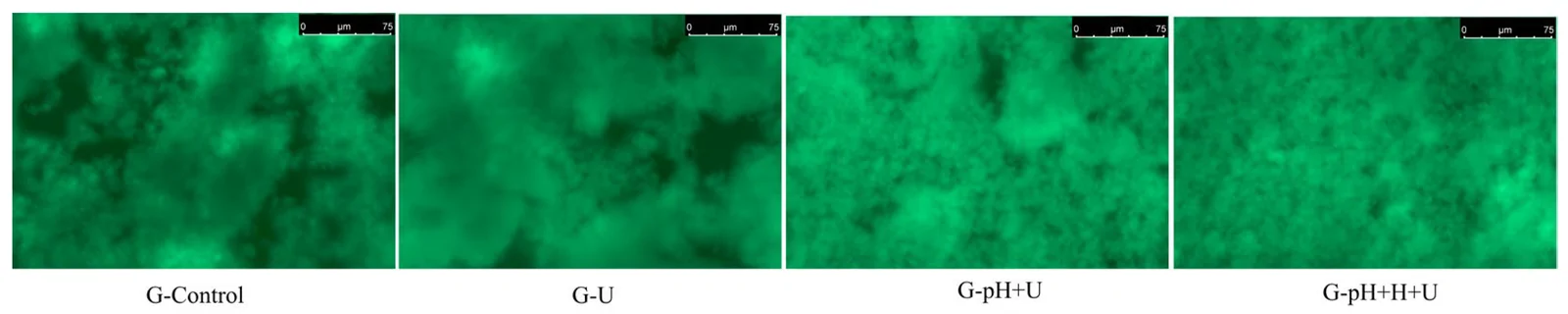
Microstructural morphology of perilla seed protein gels visualized by fluorescence microscopy following high-pressure homogenization, pH-shifting, and ultrasound treatments. G-U, G-pH+U, and G-pH+H+U represent protein gels prepared by different treatments, respectively. Treatment labels are the same as in Figure 1.

Figure 10 reveals a marked improvement in the continuity of the PSPI gel network following the modification treatments. In particular, gels prepared with PSPI modified by all three methods exhibited largely eliminated pores, leading to a denser, more compact, and uniform microstructure. These microstructural observations are consistent with the superior water-holding capacity, gel strength, viscosity, and storage modulus exhibited by the triply modified gels. Earlier studies have similarly linked enhanced intermolecular interactions to the formation of more continuous and dense gel networks with greater mechanical strength [43].

**Figure 11:**
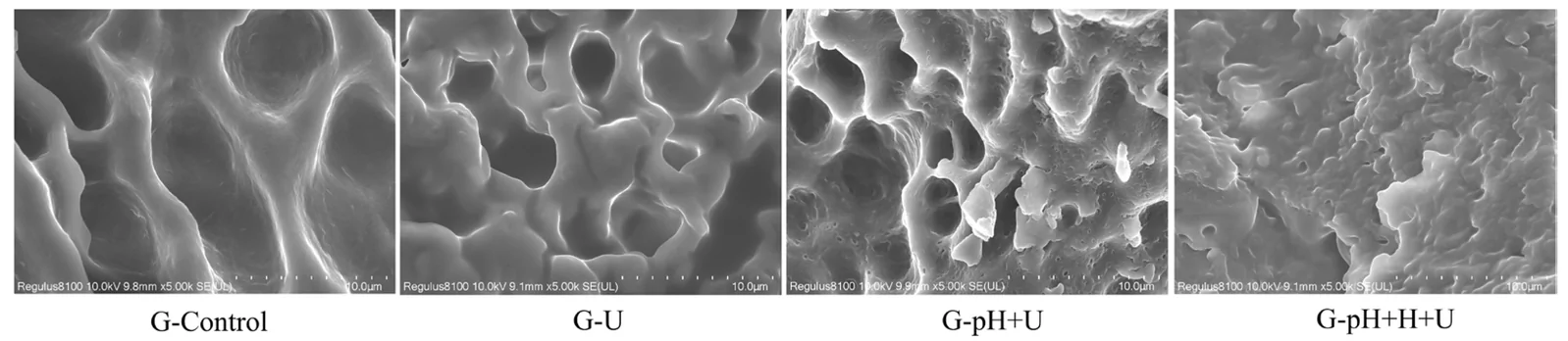
Microstructural features of perilla seed protein gels revealed by scanning electron microscopy (SEM) after high-pressure homogenization, pH-shifting, and ultrasound. G-U, G-pH+U, and G-pH+H+U represent protein gels prepared by different treatments, respectively. Treatment labels are the same as in Figure 1.

The microstructural features of these gels were further investigated using scanning electron microscopy (SEM). Figure 11 shows that the unmodified PSPI gel presented a coarse architecture composed of sheet-like aggregates. In contrast, the modified gels displayed a more homogeneous and tightly packed network with appreciably reduced pore sizes. While the presence of smaller pores is often associated with a denser network [51], we acknowledge that direct evidence of increased cross-linking density cannot be derived solely from SEM images. Nevertheless, these microstructural observations are consistent with the hypothesis that enhanced intermolecular forces, such as hydrophobic interactions or disulfide bonds (as suggested by the increased surface hydrophobicity and free sulfhydryl content of the modified proteins), may have contributed to a more compact network. Moreover, the more ordered structure may reinforce capillary forces within the gel matrix, thereby immobilizing water and restricting its movement. This observation rationalizes the higher water-holding capacity exhibited by the modified PSPI gels.

## 3. Conclusions

In this study, the individual and combined effects of ultrasound, pH-shifting, and high-pressure homogenization on the structural and gelation properties of perilla seed protein, which is a previously underexplored protein resource from medicinal and edible plants, were systematically investigated. Among all tested treatments, the most pronounced effects were exhibited by the triple combination, as evidenced by a 129% increase in solubility, the smallest particle size, the highest random coil content, and maximal surface hydrophobicity and free sulfhydryl content. These structural rearrangements were translated into superior gelation performance upon probiotic fermentation, which was reflected in enhanced gel strength, water-holding capacity, viscosity, storage modulus, and viable counts, along with a dense and uniform microstructure. We propose that these improvements are likely attributable to a combination of enhanced hydrophobic interactions and disulfide-bond formation, although direct evidence for these specific interactions in the final gel network remains to be established in future studies.

Collectively, our findings demonstrate that multi-physical modification is an effective strategy for upgrading underutilized plant proteins into functional ingredients suitable for fermented plant-protein gel applications. However, several experimental limitations should be acknowledged when interpreting these results. First, all structural and physicochemical characterizations were performed on technical replicates derived from a single batch of PSPI. While these measurements provide reliable intra-batch precision, they do not permit statistical generalization of treatment effects to independent protein preparations. In contrast, all gel property evaluations were based on three independent experimental replicates, and the statistical comparisons reported for those properties are robust. Second, because the detailed structural and gelation characterizations were carried out only on treatment sequences selected via solubility screening (U, pH+U, and pH+H+U), our experimental design does not allow the independent contributions of pH-shifting or high-pressure homogenization, nor their pairwise or three-way interactions, to be quantitatively deconvoluted. The observed progressive improvements across the selected sequences should therefore be interpreted as ordinal enhancements rather than as evidence for specific additive or synergistic effects.

Future work employing a full factorial design encompassing all treatment combinations will be necessary to delineate the individual and interactive roles of each processing parameter. Additionally, further studies are warranted to elucidate the underlying fermentation mechanisms, track real-time gelation dynamics, and evaluate sensory attributes and digestive stability, all of which are critical for facilitating industrial translation. Nonetheless, the present work provides a proof of concept that a rationally designed sequential modification protocol can effectively upgrade PSPI for fermented plant-based gel applications.

## 4. Materials and Methods

### 4.1. Materials and Chemicals

Perilla (*Perilla frutescens* (L.) Britt.) seeds were supplied by Jiangsu Chengkai Traditional Chinese Medicine Co., Ltd. (Huaian, Jiangsu, China). Shanghai Yuanye Bio-Technology Co., Ltd. (Shanghai, China) provided the fluorescein isothiocyanate (FITC) and 8-anilino-1-naphthalenesulfonic acid (ANS). The starter cultures (containing *Lactobacillus acidophilus*, *Lactobacillus bulgaricus*, *Lactobacillus plantarum*, *Lactobacillus rhamnosus*, *Streptococcus thermophilus*, *Lactobacillus casei*, and *Bifidobacterium*) were sourced from Qingdao Kaimaisen Food Technology Co., Ltd. (Qingdao, Shandong, China). Other reagents, including dimethyl sulfoxide (DMSO), o-phthalaldehyde (OPA), and Folin-phenol reagent, as well as general chemicals, were procured from Sinopharm Chemical Reagent Co., Ltd. (Shanghai, China).

### 4.2. Preparation of Perilla Seed Protein Isolate (PSPI)

PSPI was prepared by alkaline extraction followed by isoelectric precipitation [52]. In brief, perilla seeds were mechanically pulverized and defatted using n-hexane extraction. The extraction was performed at room temperature for 6 h under constant stirring with a solid-to-solvent ratio of 1:3 (*w*/*v*), and this defatting step was conducted in triplicate for thorough oil elimination. The obtained defatted meal was air-dried overnight and milled to pass through a 100-mesh sieve. Subsequently, the defatted powder was mixed with distilled water at a solid–liquid ratio of 1:10 (*w*/*v*), and the pH value was brought to 9.0 using 2 M NaOH. After 2 h of stirring at ambient temperature, the suspension was centrifuged at 6000× *g* for 25 min at 4 °C. The collected supernatant was acidified to pH 4.3 with 2 M HCl for protein precipitation. Following another centrifugation step (6000× *g*, 25 min, 4 °C), the resultant pellet was resuspended in distilled water, neutralized to pH 7.0 with 2 M NaOH, and lyophilized. The Kjeldahl method (N × 6.25) determined that the resulting PSPI powder possessed a protein content of 87.6 ± 1.6% on a dry-weight basis, with an extraction yield of 19.1 ± 0.9 g PSPI per 100 g defatted meal. The PSPI powder was preserved at 4 °C prior to subsequent experiments.

### 4.3. Modifications of PSPI

PSPI dispersions (1% *w*/*v*) were subjected to three modifications, alone or in combinations. Ultrasound (U): ultrasonic processing was carried out with an ultrasonic processor (JY92-IIDN, Ningbo Scientz Biotechnology Co., Ltd., Ningbo, Zhejiang, China) fitted with a 6 mm-diameter titanium alloy probe. A constant frequency of 20 kHz and nominal power of 400 W were applied, and the amplitude was adjusted to 40% of full output. Accordingly, 100 mL PSPI dispersion (1%, *w*/*v*) was transferred into a 250 mL glass beaker, followed by sonication for 20 min under pulsed operation (2 s on/2 s off) to avoid overheating of the sample. An ice-water bath was employed to keep the sample’s temperature at 4 °C throughout the whole process, and the probe was submerged 2 cm beneath the liquid surface [8]. The actual acoustic energy density delivered to the sample was not measured in this study; therefore, it could not be determined. pH-shifting (pH): adjusted to pH 12.0 (2 M NaOH) for 1 h, then neutralized to pH 7.0 [53]. High-pressure homogenization (H): high-pressure homogenization was carried out using a high-pressure homogenizer (SCIENTZ-Nano, Ningbo Xinzhi Biotechnology Co., Ltd., Ningbo, China) [54]. PSPI dispersion (1%, *w*/*v*) was homogenized at 90 MPa for three successive cycles. The sample was pre-cooled to 4 °C before each pass, and the outlet temperature was instantly measured by a digital thermometer; the temperature elevation throughout homogenization never exceeded 25 °C. Between cycles, the sample was fast-cooled in an ice-water bath to 4 °C to limit thermal denaturation. For each cycle, the dispersion was introduced to the homogenizer inlet, treated at 90 MPa, collected at the outlet, and rapidly cooled prior to the next run; the above operation was repeated for three passes in total. For combined treatments, the modifications were applied sequentially in the order indicated by the sample code: pH+U (pH-shifting followed by ultrasound), H+U (high-pressure homogenization followed by ultrasound), pH+H (pH-shifting followed by high-pressure homogenization), and pH+H+U (pH-shifting, followed by high-pressure homogenization, followed by ultrasound). All samples were freeze-dried. Untreated PSPI served as the control. Samples were designated U, pH, H, pH+U, H+U, pH+H, and pH+H+U.

### 4.4. Solubility of PSPI

To determine protein solubility, lyophilized PSPI samples (2 g) were first suspended in 98 mL of phosphate buffer (10 mM, pH 7.0). The resulting dispersion was then centrifuged at 10,000× *g* for 30 min at 4 °C [2]. Protein concentration in the supernatant was determined colorimetrically using a Folin-phenol assay kit, with procedures carried out in accordance with the manufacturer’s protocol (Nanjing Jiancheng Bioengineering Institute, Nanjing, China), using bovine serum albumin as the calibration standard.

### 4.5. Transmission Electron Microscopy (TEM) Visualization of PSPI Microstructure

A 20 mg/mL droplet of the sample was deposited onto a carbon-supported copper grid. After 30 s, a drop of 2.5% glutaraldehyde was applied to the grid and then blotted away with filter paper. The grid was subsequently air-dried for 10 min at room temperature. Micrographs were acquired using a JEOL JEM-2100Plus microscope (Japan Electron Optics Laboratory, Akishima, Tokyo, Japan) at a magnification of 15,000× [24].

### 4.6. Particle Size Distribution and Zeta Potential of PSPI

Particle size analysis of the modified PSPI dispersions (1%, *w*/*v*) was carried out by laser diffraction using a Beckman Coulter LS 13,320 analyzer (Beckman Coulter, Inc., Brea, CA, USA), with distilled water as the dispersing medium [2]. The refractive indices were set to 1.33 for the aqueous phase and 1.50 for the protein material. For zeta potential measurements, the samples were diluted with distilled water to 0.01% (*w*/*v*) and analyzed on a Malvern NanoZS Zetasizer (Malvern, Worcestershire, UK) [52]. The electrophoretic mobility data were converted to zeta potential values using the Henry equation.

### 4.7. Subunits of PSPI

Protein subunit composition was analyzed by SDS-PAGE using a Bio-Rad Mini-Protein Tetra system (Hercules, CA, USA) with a 12% separating gel and a 5% stacking gel [8]. A pre-stained protein ladder (Thermo Scientific, Shanghai, China) was co-electrophoresed with the samples as a molecular weight reference. The PSPI samples were prepared at a concentration of 2 mg/mL prior to loading. After electrophoresis, protein bands were visualized by staining with Coomassie Brilliant Blue R-250, and the gels were documented with a Bio-Rad GelDoc XR imaging system (Hercules, CA, USA).

### 4.8. Secondary and Tertiary Structure Analysis of PSPI

**Figure A1:**
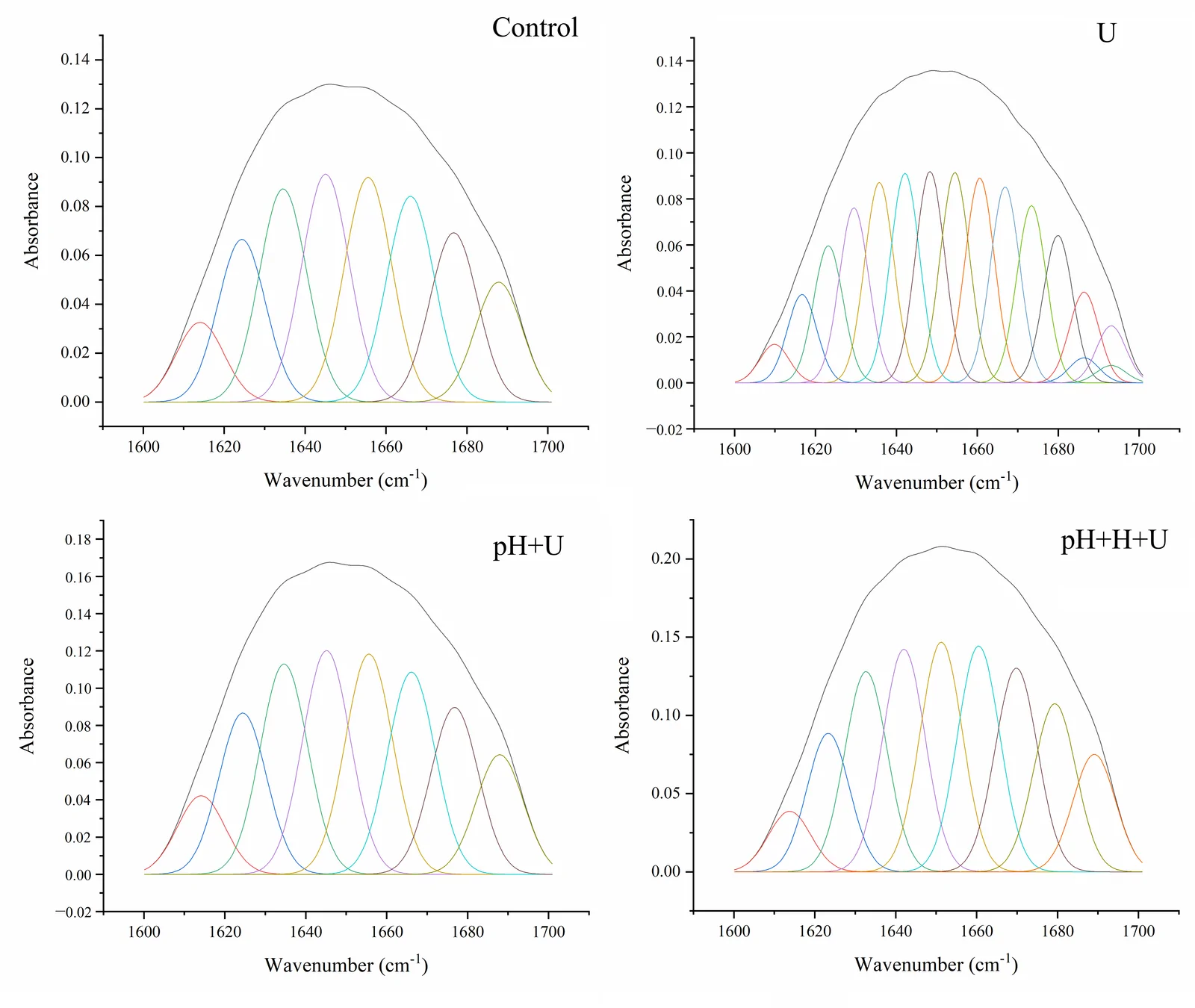
Curve-fitting analysis of Fourier self-deconvoluted amide I band from the FTIR spectra. Treatment labels are the same as in Figure 1.

The secondary structure of the protein samples was characterized by FTIR spectroscopy [2]. Spectral acquisition was performed on an FTIR-7600 spectrometer (Lambda, Australia) over the wavenumber range of 4000–400 cm^−1^. To resolve the overlapping bands within the amide I region (1600–1700 cm^−1^), Fourier deconvolution and second-derivative analysis were carried out, followed by Gaussian peak fitting (Figure A1). On the basis of the literature assignments [55], the fitted components were assigned to α-helix (1648–1664 cm^−1^), β-sheet (1615–1637 cm^−1^ and 1682–1700 cm^−1^), β-turn (1664–1681 cm^−1^), and random coil (1637–1648 cm^−1^). The content of each secondary structural element was calculated from the integrated peak areas. Tertiary structural changes were monitored by intrinsic fluorescence spectroscopy [52]. The PSPI solutions were diluted to 2 mg/mL in distilled water, and emission spectra were recorded over 300–400 nm at an excitation wavelength of 290 nm using an F380 fluorescence spectrophotometer (Tianjin Gangdong Sci. & Tech. Development Co., Ltd., Tianjin, China).

### 4.9. Surface Hydrophobicity of PSPI

For surface hydrophobicity measurement, the PSPI stock solution (2 mg/mL) was serially diluted with sulfate buffer (pH 6.86) to prepare a series of concentrations spanning 0.03 to 1.0 mg/mL. Each dilution (1 mL) was combined with 10 µL of ANS reagent (0.05%, *w*/*v*) and allowed to react. The fluorescence intensity of the mixtures was then recorded at excitation and emission wavelengths of 390 nm and 470 nm, respectively, using the same spectrofluorometer as described above. The surface hydrophobicity index was determined as the slope of the fluorescence intensity versus protein concentration plot by linear regression [8].

### 4.10. Differential Scanning Calorimetry (DSC) Tests of PSPI

Thermal transition properties of the protein samples were evaluated by differential scanning calorimetry (TA Instruments, New Castle, DE, USA). Approximately 5 mg of each sample was weighed into an aluminum pan, which was then hermetically crimped and placed in the calorimeter. The samples were heated from 30 °C to 200 °C at a constant rate of 10 °C/min under a nitrogen atmosphere. Thermograms were recorded and processed with TA Universal Analysis 2000 software (Version 4.5A).

### 4.11. Free Sulfhydryl (SH) Content

Free thiol groups were assayed using the DTNB reagent (5,5′-dithiobis-(2-nitrobenzoic acid)) [8]. Briefly, samples were reacted with DTNB for 30 min in the dark (tubes wrapped in foil) in a water bath. Absorbance was measured at 412 nm, and SH content was determined from a glutathione standard curve. Measurements were made with a Spark 10 M microplate spectrophotometer (Tecan, Männedorf, Switzerland) using quartz 96-well plates.

### 4.12. Gel Preparation and Characterization

#### 4.12.1. Preparation of PSPI Gels

For gel preparation, PSPI (5 g) and sucrose (5 g) were dissolved in water (90 mL), hydrated at 4 °C for 24 h, pasteurized (90 °C, 15 min), inoculated with starter culture (0.1 g, with an initial viable count of approximately 10^11^ CFU/g), and fermented at 42 °C for 10 h, then refrigerated at 4 °C for 24 h.

#### 4.12.2. Color Analysis

Gel whiteness was measured digitally [41].

#### 4.12.3. pH Determination

The pH was recorded using a calibrated meter.

#### 4.12.4. Water-Holding Capacity (WHC)

WHC was determined after centrifugation (10,000× *g*, 10 min).

#### 4.12.5. Gel Strength

Gel strength was measured using a texture analyzer (TA-XT2i; Stable Micro Systems, Godalming, Surrey, UK) by compressing 50 mm diameter gel specimens to half their original thickness with a 36-mm cylindrical plunger at a rate of 1 mm/s.

#### 4.12.6. Viable Count

Viable count was assessed by plate counting using MRS agar (pH 6.2) after serial dilution with 0.85% sterile saline. Plates were incubated at 37 °C for 48 h under anaerobic conditions, and colonies were enumerated as log10 CFU/mL of sample [41].

#### 4.12.7. Rheological Properties

Rheological properties were measured using a Discovery HR-1 rheometer (parallel plate, 60 mm diameter, 100 μm gap) at 25 °C, including steady shear (1–300 s^−1^), dynamic oscillatory (0.1–100 rad/s, 0.1% strain), and creep-recovery tests (5 Pa, 300 s each) [41].

#### 4.12.8. Microstructure

Protein components in the gel matrix were fluorescently labeled by thoroughly mixing 1 g of fresh gel with 10 μL of FITC (fluorescein isothiocyanate) solution (0.1 mg/mL in DMSO). The stained specimens were then visualized using a Leica DM2500 fluorescence microscope (Leica Microsystems GmbH, Wetzlar, Germany) at a magnification of 200×. The freeze-dried gel specimens were mounted onto aluminum stubs with conductive adhesive and sputter-coated with a gold layer prior to observation. The sputtering process was performed using an automatic gold coater with the following preset parameters: accelerating voltage 1.2 kV, sputtering current 10 mA, and sputtering time 60 s (working distance and chamber pressure were set according to the manufacturer’s default configuration). Coated samples were then examined using a scanning electron microscope (Regulus8100, Hitachi, Tokyo, Japan) at an accelerating voltage of 10 kV. Images were recorded at 5000× magnification, with a working distance of 9–10 mm and an emission current of 10 µA under high vacuum (1 × 10^−4^ Pa).

### 4.13. Statistical Analyses

This study involved two types of replicates depending on the stage of analysis. For protein structural and physicochemical characterization, all measurements were performed in triplicate using the same independently prepared batch of PSPI. These technical replicates were used to assess measurement precision, and the data are presented as mean ± SD. No statistical inference was applied to these structural/physicochemical data, as technical replicates from a single batch do not constitute independent experimental units for treatment-effect inference. Observed differences are discussed descriptively in terms of trend. For gel preparation and gel property analyses, the experiments were conducted using three independent experimental replicates. The resulting data were subjected to one-way analysis of variance (ANOVA) using SPSS for Windows (version 17.0, Tallahassee, FL, USA). When a significant main effect was detected (*p* < 0.05), mean comparisons were performed using Tukey’s honestly significant difference (HSD) post hoc test to identify which specific groups differed from each other. Results are expressed as mean ± standard deviation (SD). A completely randomized linear model was adopted for the ANOVA. Statistical significance was set at a 95% confidence level, and differences with a *p*-value below 0.05 were considered significant.

## Data Availability

The raw data supporting the conclusions of this article will be made available by the authors on request.

## Abbreviations

PSPI	Perilla seed protein isolate
FITC	Fluorescein isothiocyanate
ANS	8-anilino-1-naphthalenesulfonic acid
U	Ultrasound
pH	pH
H	High-pressure homogenization
TEM	Transmission electron microscopy
FTIR	Fourier transform infrared spectroscopy
DSC	Differential scanning calorimetry
SEM	Scanning electron microscopy
